# Drug-Coated Balloon versus Drug-Eluting Stent in Patients with Small-Vessel Coronary Artery Disease: A Meta-Analysis of Randomized Controlled Trials

**DOI:** 10.1155/2021/1647635

**Published:** 2021-04-13

**Authors:** Xinying Wu, Lun Li, Li He

**Affiliations:** Department of Cardiology, Wuhan Fourth Hospital, Puai Hospital, Tongji Medical College, Huazhong University of Science and Technology, Han Zheng Street 473, Wuhan 430033, China

## Abstract

**Background:**

Percutaneous coronary intervention (PCI) with drug-eluting stents (DES) of small-vessel coronary artery disease (SVD) is related to an increased risk of in-stent restenosis (ISR) and stent thrombosis (ST). The application of the drug-coated balloon (DCB) for patients with SVD remains controversial.

**Objectives:**

Assess the outcomes of DCB in the treatment of SVD compared with DES in patients with SVD.

**Methods:**

A meta-analysis of randomized controlled trials (RCTs) published up to June 2020, reporting the outcomes of DCB versus DES in the treatment of SVD, was performed.

**Results:**

Four RCTs with 1227 patients were included. The results indicated that DCB was associated with the decreased risk for myocardial infarction (MI) compared with the DES, but the difference showed no significance (OR 0.50, 95% CI 0.24–1.03, *P*=0.06). And, there was no significant difference in death (OR 0.76, 95% CI 0.17–3.43, *P*=0.72), cardiac death (OR 1.92, 95% CI 0.74–4.98, *P*=0.18), target vessel revascularization (TVR) (OR 0.81, 95% CI 0.51–1.28, *P*=0.36), target lesion revascularization (TLR) (OR 1.29, 95% CI 0.66–2.52, *P*=0.46), and major adverse cardiac events (MACE) (OR 0.92, 95% CI 0.61–1.38, *P*=0.69) between the DCB group and DES group.

**Conclusion:**

Compared with DES, DCB was associated with a decreased risk of MI among patients with SVD, but the difference showed no significance. The application of DCB in SVD is associated with comparable outcomes of death, TVR, and MACE when compared with DES.

## 1. Introduction

Drug-eluting stents (DES) were normative of care for severe coronary artery disease (CAD). However, the efficacy of DES was limited by stent thrombosis (ST) and in-stent restenosis (ISR) [1]. The drug-coated balloon (DCB) delivers the antiproliferative drug into the vessel wall without implanting a stent. The DCB was a novel therapeutic strategy to overcome the ISR of bare-metal stents (BMS) and DES and recommended in the European Society of Cardiology guidelines (class I, level of evidence A) [2, 3]. But the application of DCB for patients with SVD remains controversial because different studies have obtained conflicting conclusions. In recent studies, SVD has been identified as an angiographic reference vessel diameter of less than 3 mm as the most appropriate cutoff [4]. SVD is common among the patients who underwent PCI [5, 6], which also remains an independent predictor of major adverse cardiac events (MACE) [7]. We conducted a meta-analysis of RCTs directly to compare DCB and DES on several outcomes in patients with SVD in the hope of providing a better choice for clinical treatment.

## 2. Methods

### 2.1. Search Strategy

It was searched for PubMed, Embase, and Cochrane Library up to June 2020 to identify randomized controlled trials (RCTs). Searches were conducted without any language restrictions using the keywords “drug-eluting balloon,” “drug-coated balloon,” “drug-eluting stent,” “drug-coated stent,” “small coronary artery disease,” “small coronary vessel,” “small vessel coronary artery disease,” and “small vessel disease.” The electronic search strategy was complemented by a manual review of the reference list of each included article. The report of the methods in this meta-analysis was in accord with Preferred Reporting Items for Systematic Reviews and Meta-Analyses guidelines [8]. All analyses were based on published RCTs; therefore, no ethical approval or patient consent was required.

### 2.2. Study Selection

The inclusion criteria in this meta-analysis were (1) all patients with SVD, (2) complete reporting of clinical outcomes such as myocardial infarction (MI), death, cardiac death, target vessel revascularization (TVR), target lesion revascularization (TLR), and major adverse cardiac events (MACE), (3) comparison of the DCB and DES strategies, and (4) randomized controlled trials (RCTs). Studies lack comparison or control groups, in which data concerning the above outcomes were excluded.

### 2.3. Data Extraction and Quality Assessment

Data extraction was performed by two authors using a standardized data collection form, and disagreements were resolved by discussion. Patient characteristics, study quality, and clinical outcomes, including MI, death, cardiac death, TVR, TLR, and MACE, were analyzed in both the DCB group and DES group.

The eligible studies' risk of bias was assessed using the Cochrane collaboration's tool for randomized controlled trials [9]. The tool consists of 7 points: random sequence generation, allocation concealment, blinding of participants and personnel, blinding of outcome assessment, incomplete outcome data, selective reporting, and other biases. Trials with >2 high-risk components were considered a moderate risk of bias, and trials with >4 high-risk components were considered a high risk of bias.

### 2.4. Statistical Analysis

The statistical analyses were performed using the Review Manager (RevMan) software version 5.3. Count data were expressed as odds ratios (ORs) and 95% confidence intervals (CIs). Statistical heterogeneity tested was performed using the value of *P* and *I*^2^ when the heterogeneity test showed *P* ≥ 0.1, and *I*^2^ ≤ 50% was considered to have homogeneity; a fixed-effects model was selected. Otherwise, when the heterogeneity test showed *P* < 0.1, *I*^2^ > 50% was considered to have substantial heterogeneity, and a random-effects model was used.

## 3. Results

### 3.1. Search Results

**Figure 1:**
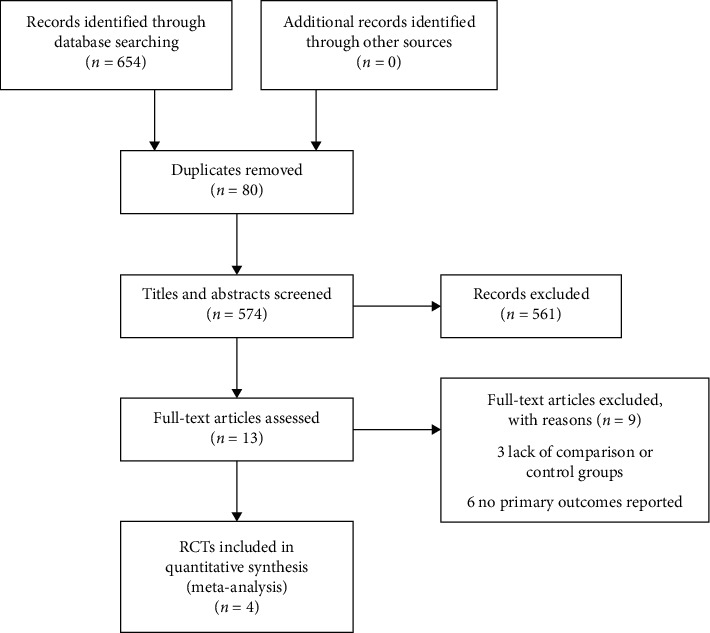
Flow diagram of the study selection process.

The search results are shown in Figure 1. The initial search retrieved 654 articles, of which 80 were duplicates. After browsing titles, abstracts, and full texts, the final 4 RCTs [10–15] were included in this meta-analysis.

### 3.2. Study Characteristics

The baseline characteristics of the included RCTs are presented in Table 1. Four RCTs containing 1227 patients with SVD were eligible for inclusion in the meta-analysis. Patients in the DCB group received DIOR, IN.PACT Falcon, SeQuent Please, or RESTORE paclitaxel-coated balloons, while those in the DES group received the first or second-generation DES from different manufacturers. The outcomes of the included RCTs are demonstrated in Table 2.

### 3.3. Quality Assessment and Risk of Bias

**Figure 2:**
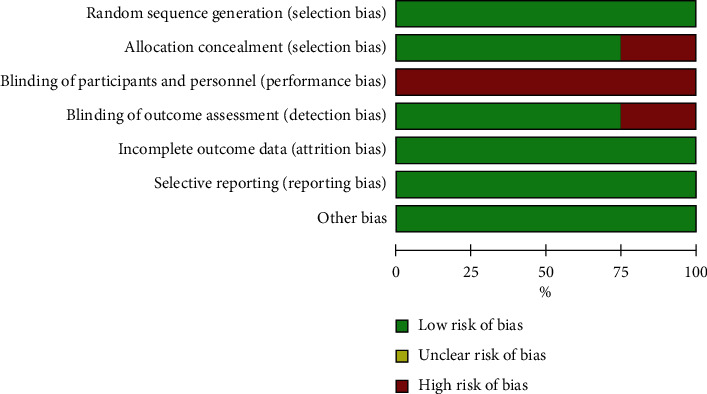
Risk of bias graph.

**Figure 3:**
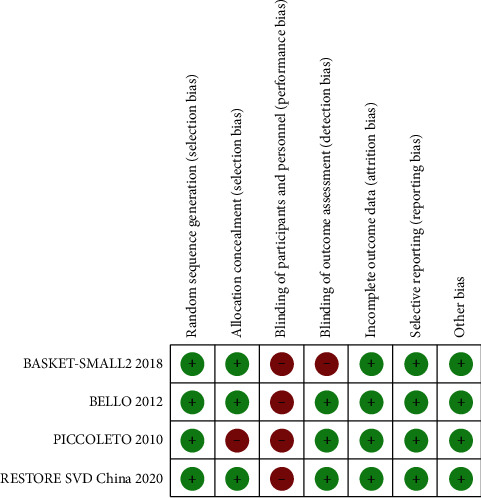
Risk of bias summary.

The quality of most RCTs was higher, according to the Cochrane quality assessment criteria (Figures 2 and 3). The whole four RCTs revealed a high risk of bias at the blinding of participants and personnel. The BASKET-SMALL2 study and PICCOLETO study showed a high risk of bias at blinding outcome assessment and allocation concealment.

### 3.4. Meta-Analysis Results

**Figure 4:**
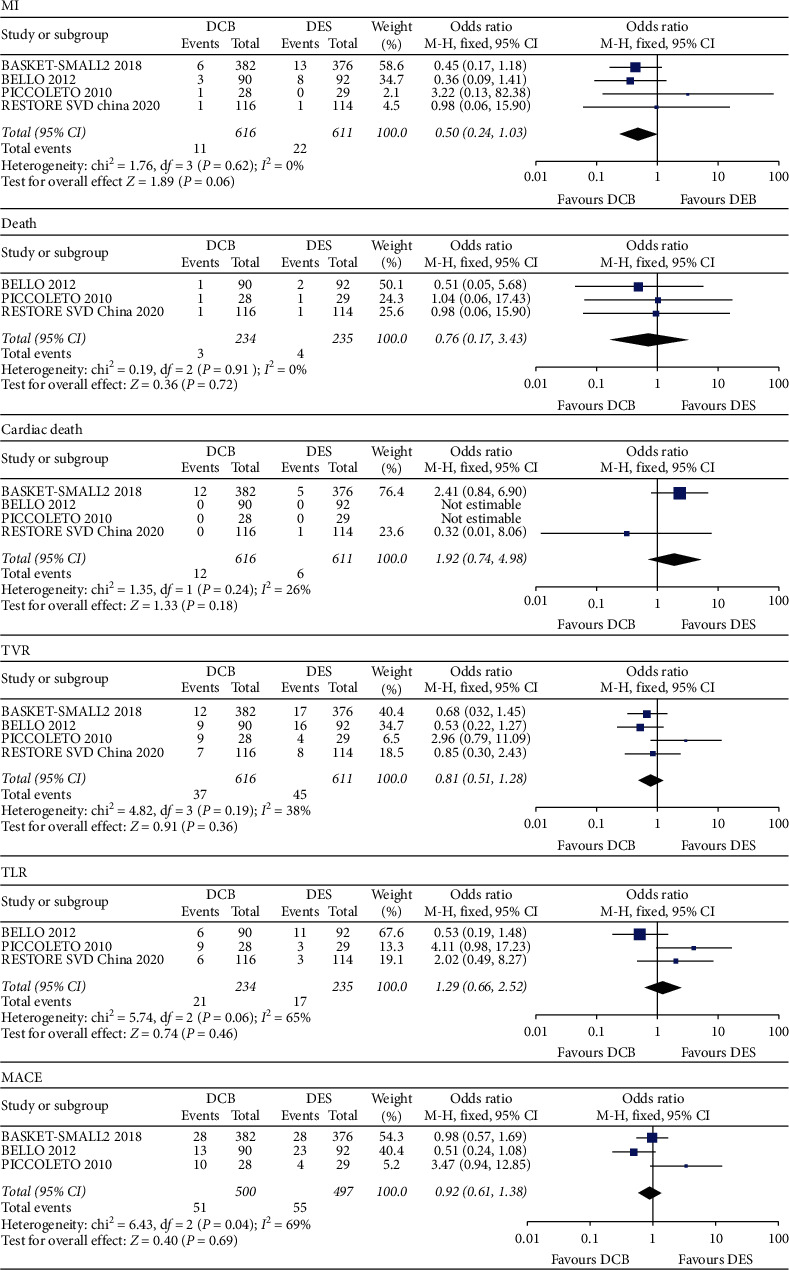
Forest plots comparing DCB with DES in MI, death, cardiac death, TVR, TLR, and MACE. DCB, drug-coated balloon; DES, drug-eluting stent; MACE, major adverse cardiac events; MI, myocardial infarction; TLR, target lesion revascularization; TVR, target vessel revascularization.

The results indicated that compared with the DES, DCB was associated with the decreased risk of MI in patients with SVD, but the difference showed no significance (OR 0.50, 95% CI 0.24–1.03, *P*=0.06). And, there was no significant difference in death (OR 0.76, 95% CI 0.17–3.43, *P*=0.72), cardiac death (OR 1.92, 95% CI 0.74–4.98, *P*=0.18), TVR (OR 0.81, 95% CI 0.51–1.28, *P*=0.36), TLR (OR 1.29, 95% CI 0.66–2.52, *P*=0.46), and MACE (OR 0.92, 95% CI 0.61–1.38, *P*=0.69) between the DCB group and DES group (Figure 4).

## 4. Discussion

Currently, PCI with DES has been widely promoted for patients with CAD. Given the limitations of DES in terms of ISR and ST, DCB angioplasty has shown to be a new strategy for the treatment of de novo stenosis in SVD. Only a few RCTs compared DCB angioplasty with DES in SVD. In this meta-analysis, the efficacy of DCB and DES in patients with SVD was compared. The results indicated that the DCB strategy was associated with decreased risk for MI compared with the DES strategy, but there was no significant difference. And there were no statistical differences in the other clinical outcomes such as all-cause death, cardiac death, TVR, TLR, and MACE, which were consistent with the conclusion of the earlier study reported by Li et al. [16].

PCI aims to improve the minimum lumen diameter in a given target coronary segment, which has a specific reference vessel diameter [17]. Thus, the minimum lumen diameter increases after the procedure but decreases at follow-up, mainly because of hyperplasia phenomena and recoil. DES implantation results in arterial injury, initiating a vascular-proliferative cascade with smooth muscle cell proliferation and migration, resulting in neointimal hyperplasia. Thus, the amount of neointimal hyperplasia is mainly independent of vessel size. Small vessels were easier to restenosis than larger vessels [18, 19]. DCB delivers the antiproliferative drug into the vessel wall without implanting a stent that can play a suppression role of the intimal hyperplasia after it contacts the vessel wall, thereby reducing the inflammation of the intima.

PICCOLETO trial is the first RCT to compare DCB (DIOR paclitaxel-coated balloon) and DES (Taxus Liberté paclitaxel-eluting stent) in SVD [10]. The study was terminated early after the inclusion of 57 patients based on an interim analysis which showed a higher rate of target lesion stenosis after six months (DCB 44% vs. DES 24%, *P*=0.029) and a higher risk of MACE (36% in the DCB group vs. 14% in the DES group, *P*=0.054). The principal investigator of the PICCOLETO trial hypothesized these findings were based on a lack of efficacy of the DIOR paclitaxel-coated balloon, which they used, which was later replaced by newer generation DCBs [20].

BELLO study [11] randomized 182 patients with SVD 1 : 1 to paclitaxel-coated balloon (IN.PACT Falcon) group and paclitaxel-eluting stent (Taxus Liberté) group. The clinical efficacy of DCB angioplasty was confirmed for up to 2 years, showing a trend toward improved outcomes with regard to MACE (DCB 14.8% vs. DES 25.3%; *P*=0.08).

Balloon predilatation is essential, which can be seen in the BELLO trial; balloon predilatation was performed in 96.8% of interventions, while 25% in the PICCOLETO study [1]. In the following BASKET-SMALL2 and RESTORE SVD China studies, after successful balloon predilatation, DCB indicated not inferior to new generation DES on cardiac death, MI, and TVR in 1-2 years follow-up [13–15].

**Figure 5:**
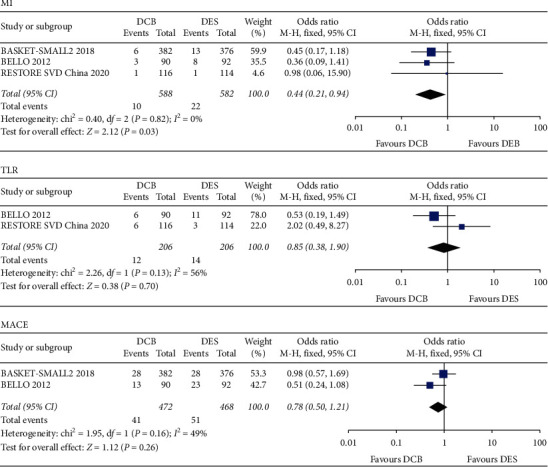
Forest plots comparing DCB with DES in MI, TLR, and MACE (excluded PICCOLETO trial). MACE, major adverse cardiac events; MI, myocardial infarction; TLR, target lesion revascularization.

If the data of the PICCOLETO study were excluded from this meta-analysis, it would be found that the DCB strategy was associated with a significant reduction in the clinical outcomes of MI (OR 0.44, 95% CI 0.21–0.94, *P*=0.03), and the heterogeneity of TLR and MACE would be improved (Figure 5). Tailored DCB device selection and sufficient balloon predilatation were helpful to the outcomes of PCI with DCB in patients with SVD.

Furthermore, we speculate no difference in outcome between the two strategies, which may be related to the multiple pathophysiological pathways involved in ischemic heart disease and restenosis after PCI. Mechanisms of ischemic heart disease are complicated, including microvascular dysfunction, inflammatory response, atherosclerotic plaque rupture, and vasospasm [21]. And multiple factors, such as biological, genetic, mechanical, and technical, may contribute to DES restenosis [22]. Compared with DES, DCB was associated with a decreased risk of stent under expansion, stent fracture, polymer damage, and stent gap. But inflammation, neoatherosclerosis, and genetic factors might also play a critical role in DCB as well as DES restenosis.

Some limitations of this meta-analysis should also be mentioned. First, the duration of the follow up of included RCTs was short. Second, the lack of extensive RCTs limits the power to detect differences between the outcomes of the compared groups. Finally, only paclitaxel-coated balloons were used in the included RCTs. Current, sirolimus-coated balloons are available and may have promising results [23].

## 5. Conclusions

In summary, this meta-analysis found that DCB was associated with decreased risk for MI compared with DES among patients with SVD, but the difference showed no significance. The application of DCB in SVD is associated with comparable outcomes of death, cardiac death, TVR, TLR, and MACE when compared with DES.

## Figures and Tables

**Table 1:** Main characteristics of included studies.

Study	PICCOLETO [10]	BELLO [11, 12]	BASKET-SMALL2 [13]	RESTORE SVD China [14, 15]
Year	2010	2012	2018	2020
DCB/DES patients (*n*/*n*)	28/29	90/92	382/376	116/114
Clinical follow-up time (months)	9	24	12	24
Definition of SVD, in diameter (mm)	≤2.75 mm	<2.8 mm	<3 mm in diameter	≥2.25 and ≤2.75 mm
Outcomes	MACE, death, MI, TLR	MACE, death, MI, TVR	MACE, cardiac death, MI, TVR	TLF, cardiac death, MI, TVR
Type of DCB	Paclitaxel-coated balloon (Dior, Eurocor GmBH, Germany)	Paclitaxel-coated balloon (IN.PACT Falcon, Medtronic, USA)	Paclitaxel-coated balloon (SeQuent Please, B Braun Melsungen AG, Germany)	Paclitaxel-coated balloon (RESTORE, Cardionovum, Germany)
Type of DES	First-generation paclitaxel-eluting stent (Taxus Liberté, Boston Scientific, USA)	First-generation paclitaxel-eluting stent (Taxus Liberté, Boston Scientific, USA)	everolimus-eluting stent (Xience, Abbott vascular, USA) or the second-generation paclitaxel-eluting stent (Taxus element, Boston Scientific, USA)	zotarolimus-eluting stent (RESOLUTE Integrity, Medtronic, USA)
Balloon predilation in the DCB group (%)	25	96.8	73	100

DCB, drug-coated balloon; DES, drug-eluting stent; MACE, major adverse cardiac events; MI, myocardial infarction; TLF, target lesion failure; TLR, target lesion revascularization; TVR, target vessel revascularization; SVD, small vessel disease. MACE definition: PICCOLETO: death, new ST-elevation MI, and TLR; BELLO: death, Q- or non-Q-wave MI, or TVR; BASKET-SMALL2: cardiac death, non-fatal MI, and TVR.

**Table 2:** Outcomes of included studies.

Study	PICCOLETO [10]		BELLO [11, 12]		BASKET-SMALL2 [13]		RESTORE SVD China [14, 15]	
	DCB (*n* = 28)	DES (*n* = 29)	DCB (*n* = 90)	DES (*n* = 92)	DCB (*n* = 382)	DES (*n* = 376）	DCB (*n* = 116)	DES (*n* = 114)
MACE (%)	10 (35.7)	4 (13.8)	13 (14.8)	23 (25.3)	28 (7.3)	28 (7.5)	N/A	N/A
Death (%)	1 (3.6)	1 (3.5)	1 (1.1)	2 (2.2)	N/A	N/A	1 (0.9)	1 (0.9)
Cardiac death (%)	0	0	0	0	12 (3.1)	5 (1.3)	0	1 (0.9)
MI (%)	1 (3.6)	0	3 (3.4)	8 (8.8)	6 (1.6)	13 (3.5)	1 (0.9)	1 (0.9)
TLR (%)	9 (32.1)	3 (10.3)	6 (6.8)	11 (12.1)	N/A	N/A	6 (5.2)	3 (3.8)
TVR (%)	9 (32.1)	4 (13.8)	9 (10.2)	16 (17.6)	12 (3.4)	17 (4.5)	7 (6.1)	8 (7.3)
TLF (%)	N/A	N/A	N/A	N/A	N/A	N/A	6 (5.2)	4 (3.7)

DCB, drug-coated balloon; DES, drug-eluting stent; MACE, major adverse cardiac events; MI, myocardial infarction; TLF, target lesion failure; TLR, target lesion revascularization; TVR, target vessel revascularization; SVD, small vessel disease. MACE definition: PICCOLETO: death, new ST-elevation MI, and TLR; BELLO: death, Q- or non-Q-wave MI, or TVR; BASKET-SMALL2: cardiac death, non-fatal MI, and TVR.
